# An Update on the Multifaceted Role of NF-kappaB in Endometriosis

**DOI:** 10.7150/ijbs.72707

**Published:** 2022-07-04

**Authors:** Yuanmeng Liu, Jianzhang Wang, Xinmei Zhang

**Affiliations:** 1 Department of Gynecology, Women's Hospital, School of Medicine, Zhejiang University, Xueshi Road, Hangzhou 310006, China; 2 Zhejiang Provincial Key Laboratory of Precision Diagnosis and Therapy for Major Gynecological Diseases, Women's Hospital, Zhejiang University School of Medicine, Hangzhou 310006, China

**Keywords:** NF-κB, endometriosis, peritoneal macrophage

## Abstract

Endometriosis remains a common but challenging gynecological disease among reproductive-aged women with an unclear pathogenesis and limited therapeutic options. Numerous pieces of evidence suggest that NF-κB signaling, a major regulator of inflammatory responses, is overactive in endometriotic lesions and contributes to the onset, progression, and recurrence of endometriosis. Several factors, such as estrogen, progesterone, oxidative stress, and noncoding RNAs, can regulate NF-κB signaling in endometriosis. In the present review, we discuss the mechanisms by which these factors regulate NF-κB during endometriosis progression and provide an update on the role of NF-κB in affecting endometriotic cells, peritoneal macrophages (PMs) as well as endometriosis-related symptoms, such as pain and infertility. Furthermore, the preclinical drugs for blocking NF-κB signaling in endometriosis are summarized, including plant-derived medicines, NF-κB inhibitors, other known drugs, and the potential anti-NF-κB drugs predicted through the Drug-Gene Interaction Database. The present review discusses most of the studies concerning the multifaceted role of NF-κB signaling in endometriosis and provides a summary of NF-κB-targeted treatment in detail.

## Introduction

Endometriosis is a benign gynecological disease that affects 6%-10% of reproductive-aged women and 20%-50% of women with infertility 1, 2. It is defined as the abnormal implantation of the endometrium outside the uterine cavity, particularly in the ovaries and pelvic peritoneum 3, 4. The colonization and growth of ectopic endometrium can result in chronic pelvic pain, infertility, dysmenorrhea, and other clinical symptoms in endometriosis patients 3, 4. Increased inflammatory responses in ectopic endometrial tissues are believed to be strongly associated with the pathogenesis of endometriosis, which is induced by the activation of proinflammatory factors and signaling pathways, as well as the increased infiltration of immune cells 5-7.

**Figure 1:**
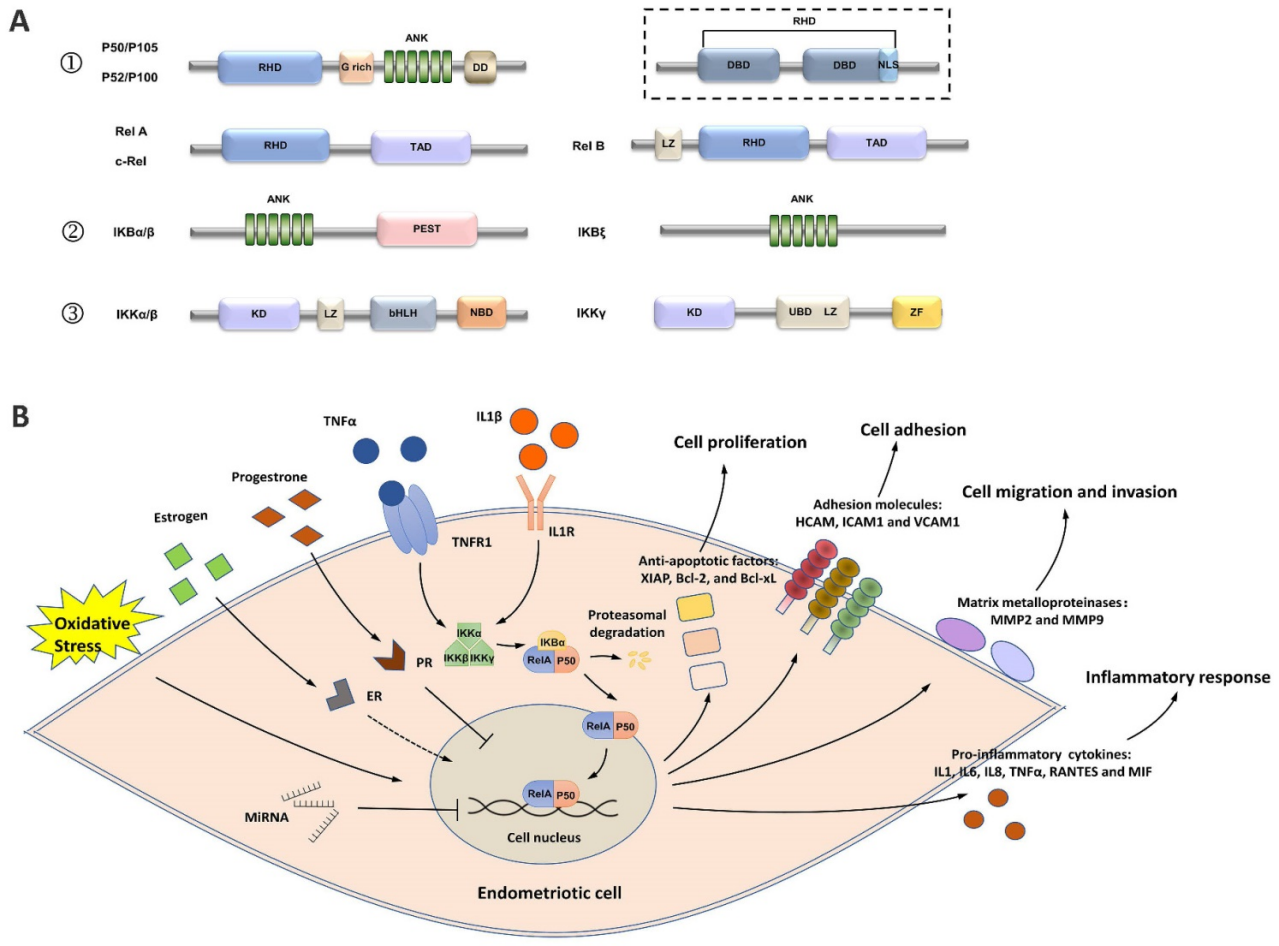
A simplified view of the structure and transduction process of NF-κB. **(A)** Structures of the members of the NF-κB signaling pathway. ① The NF-κB superfamily contains five members: RelA (p65), RelB, c-Rel, NF-κB1 (p105/p50), and NF-κB2 (p100/p52). p50 and p52 are shorter forms processed by p105 and p100, respectively. All members share a Rel homology domain (RHD), which contains two specific DNA-binding domains (DBDs) and a nuclear localization sequence. One of the DBDs is engaged in DNA recognition, and the other is involved in dimerization. ② The IκB family contains the three most important members: IKBα, IKBβ, and IKBγ. ③ The IKK complex contains the three most important members. GRR: glycine-rich region; ANK: ankyrin repeats; DD: death domain; TAD: transcription activation domain; LZ: leucine zipper domain; KD: kinase domain; HLH: helix-loop-helix domain; NBD: NEMO-binding domain; MOD/UBD: minimal oligomerization domain/ubiquitin-binding domain; ZF: zinc finger domain.** (B)** NF-κB signaling is activated in endometriotic cells. Stimuli, such as TNF-α and IL1, activate the IKK complex, triggering IKB phosphorylation and its subsequent proteasomal degradation. The RelA/p50 heterodimers then translocate to the nucleus and elicit transcriptional activity. Estrogen, progesterone, oxidative stress, and ncRNAs are the key regulators of NF-κB signaling, and the effects of estrogen on NF-κB signaling are controversial. Activation of the downstream genes of NF-κB signaling induces inflammatory responses and supports the survival, adhesion, migration, and invasion of endometriotic cells during endometriosis development.

The activation of nuclear factor kappa B (NF-κB) in patients with endometriosis has been found to play a vital role in regulating disease progression through complex mechanisms 8. As a superfamily of transcription factors, NF-κB has five members, including RelA (p65), RelB, c-Rel, NF-κB1 (p105/p50), and NF-κB2 (p100/p52) 9 **(Figure 1A)**. All five members share a Rel homology domain (RHD), which is essential for homo or heterodimerization and binding to cognate DNA elements 9. The resting state of NF-κB dimers is sequestered in the cytoplasm by a family of inhibitors of κB (IκB) proteins (such as IκBα, IκBβ, p105, and p100), which serve as inhibitors through their ankyrin repeats 10. When NF-κB signaling is activated, IκB is phosphorylated by IκB kinases (IKKs) and degraded by the proteasome, which allows NF-κB dimers to enter the nucleus and elicit the transcriptional activity of downstream genes 8.

In endometriotic cells, NK-κB signaling is activated by stimuli, such as tumor necrosis factor α (TNF-α) and interleukin-1β (IL-1β) 11-19. The IKK complex, which contains two catalytic subunits (IKK-1 [IKK-α] and IKK-2 [IKK-β]) and a noncatalytic accessory protein NF-κB essential modulator (NEMO [IKKγ]), is activated under stimulation **(Figure 1B)** 20. The IKK complex then phosphorylates IκB proteins, allowing the cytoplasmic RelA/p50 heterodimer to be released and translocated to the nucleus 20. The transcriptional activity of several proinflammatory cytokines/chemokines, such as IL1, IL6, IL8, TNF-α, RANTES, MIF, and ICAM1, is activated by NF-κB signaling, indicating the key role of NF-κB in the inflammatory responses in endometriosis 11, 13, 14, 18, 19, 21-27.

NK-κB signaling has a close relationship with key regulatory factors for the onset and progression of endometriosis, including estrogen, progesterone, oxidative stress, and noncoding RNAs (ncRNAs). In addition, NK-κB signaling can regulate the cellular behaviors of endometriotic cells and peritoneal macrophages (PMs) in the endometriotic milieu, as well as contribute to endometriosis-induced pain and infertility. In this review, we discuss the role of NF-κB in endometriosis pathogenesis and the relevant molecular mechanisms. We also summarize and predict known and potential anti-NF-κB drugs for endometriosis treatment.

## Regulation of NF-κB signaling in endometriosis

### Estrogen

**Figure 2:**
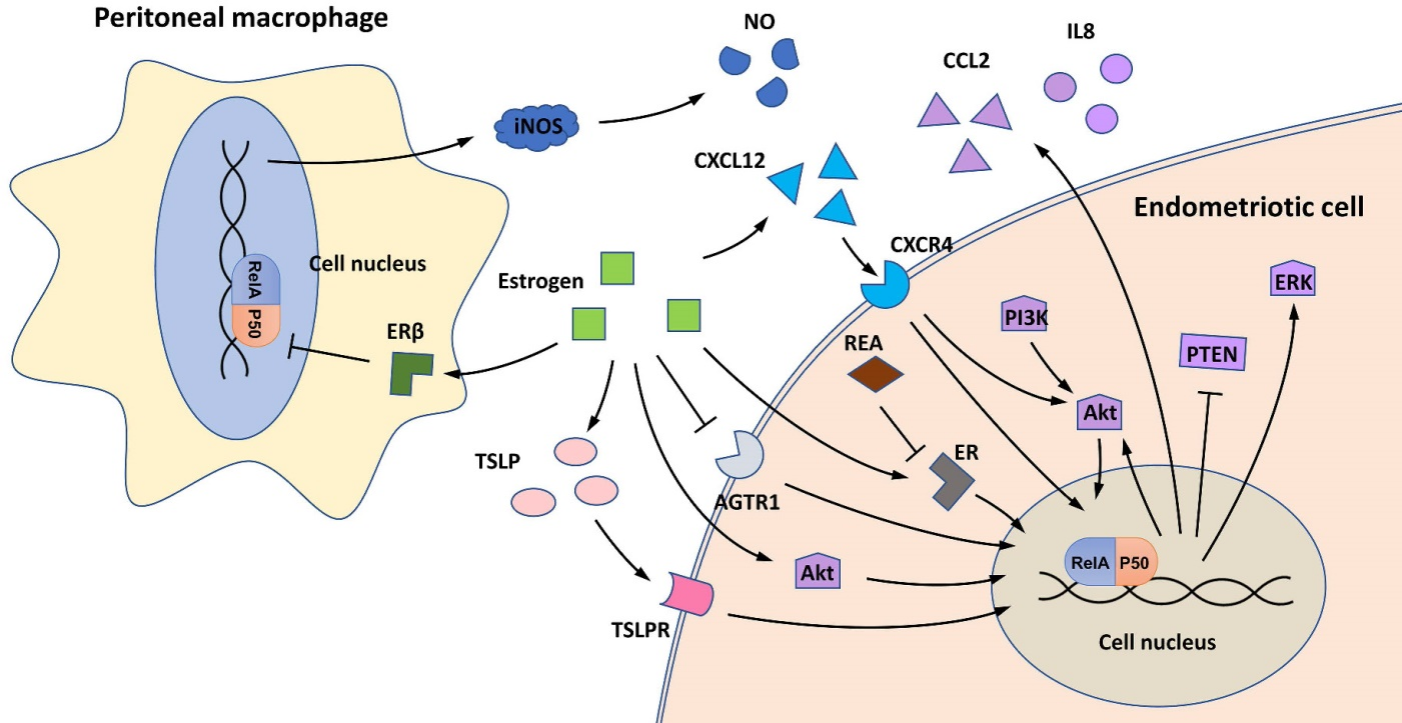
NF-κB and estrogen in endometriosis. In endometriotic cells, estrogen can activate NF-κB signaling by activating CXCL12/CXCR4, PI3K/Akt, and TSLP signaling, and low REA expression also contributes to the activation of the estrogen/NF-κB axis. Estrogen-stimulated NF-κB activity further activates Akt and ERK signaling, represses PTEN expression, and induces production of the proinflammatory cytokines CCL2 and IL8. In addition, estrogen can inhibit NF-κB signaling in endometriotic cells by repressing AGTR1 expression. In peritoneal macrophages, estrogen/ERβ signaling can inhibit NF-κB activation and further inhibit NOS production.

The effects of estrogen signaling on NF-κB in endometriosis are controversial **(Figure 2)**. An early study first discovered that estrogen and/or its receptors, estrogen receptor (ER) α and ERβ, can increase NF-κB activity in ectopic endometrial cells, contrary to their effect in normal endometrial stromal cells 28. Repressor of estrogen receptor activity (REA), a key ER corepressor in the female reproductive tract, is downregulated in ectopic endometriotic lesions, contributing to the activation of estrogen signaling and enhanced NF-κB activity 29. Mechanistically, estrogen signaling induces NF-κB activation in endometriotic cells by activating several proinflammatory pathways, such as CXCL12/CXCR4, PI3K/Akt, and thymic stromal lymphopoietin (TSLP) signaling 25, 30, 31.

Estrogen-stimulated NF-κB activation can promote the viability and proliferation of endometriotic cells. Mechanistically, NF-κB signaling can restrict autophagy-related cell death, inhibit the expression of PTEN and activate the PI3K/Akt and MAPK/ERK pathways 30, 31. In addition, estrogen-induced NF-κB activity can also affect the polarization of PMs in the endometriotic milieu (see the “Macrophage polarization” section) 32.

The treatment of endometriotic cells with GnRHa, a hormone therapy that induces the hypoestrogenic state of ectopic endometrium, reduces NF-κB activation in endometriotic cells and further suppresses the expression of the proinflammatory cytokine IL8 22. This finding indicates that hormone therapies may have the potential to inhibit NF-κB signaling and reduce inflammation in ectopic endometrial lesions 22.

However, some studies have reported the inhibitory effect of estrogen signaling on NF-κB in endometriosis 33-35. Mechanistically, estrogen can reduce the expression of *AGTR1*, a gene that encodes the angiotensin II receptor, which is overexpressed and activates NF-κB signaling in endometriosis 34. By applying a microarray-based technique, Han et al*.* found that TNFα/NF-κB signaling is downregulated by ERβ in the eutopic endometrium of a C57BL/6 mouse model 33. In addition, NF-κB signaling can also be repressed by estrogen signaling in PMs 35. Treating PMs with ERB-041, a selective ERβ agonist, inhibits NF-κB activation and its downstream inducible nitric oxide synthase (iNOS)/nitric oxide (NO) signaling during endometriosis progression 35.

SR-16234 is a selective estrogen receptor modulator that has potent antagonistic activity on ERα with weak partial agonist activity against the ERβ receptor 36. In a BALB/c mouse model of endometriosis, treatment with SR-16234 substantially decreased NF-κB p65 expression and reduced the growth of endometriotic lesions 36. In an open-label single-arm clinical trial, the oral administration of SR-16234 substantially relieved the pain symptoms of patients with endometriosis 37. These findings indicate that selectively inhibiting estrogen/ERα signaling while activating estrogen/ERβ signaling may serve as a potential therapeutic strategy for endometriosis treatment.

### Progesterone

The p65 subunit of NF-κB and progesterone receptor (PR) can repress each other through direct contact. The mutual repression of p65 and PR in the endometrium is involved in endometrial biologic alterations during the menstrual cycle and pathophysiologic processes, such as irregular uterine bleeding 38-40. In a study of 109 patients with endometriosis, increased p65 expression and decreased PRB (a PR isoform) expression jointly served as biomarkers for the recurrence of ovarian endometrioma 41. However, another study with 104 patients drew the opposite conclusion that the recurrence of ovarian endometriosis is associated with decreased p65 expression and increased PRB expression 42. The contradictory conclusions of the two reports may have been partially caused by clinical, immunological, histochemical, inflammatory, and genetic-epigenetic heterogeneity of endometriotic tissues 43-45. In addition, other factors, such as the low reproducibility of immunohistochemistry analysis, and the different patterns between recurrent and initial endometriosis, may have also affected the results 42. These findings indicate that the relationship between p65/PR expression and endometriosis recurrence needs to be re-evaluated.

As a commonly used hormone drug for treating endometriosis, progesterone inhibits the NF-κB-induced production of proinflammatory factors in endometriotic cells 46, 47, and the combined use of progesterone and NF-κB inhibitors can remarkably increase the efficacy of alleviating endometriosis-related pain 48. However, a recent study revealed that progesterone resistance, a central element during endometriosis progression, may weaken the inhibitory effect of progesterone on NF-κB by inducing aberrant endoplasmic reticulum stress in endometriotic tissues 49. The upregulation of endoplasmic reticulum stress in endometriotic cells by its activator can remarkably inhibit NF-κB-induced inflammation by upregulating the NF-κB-negative regulators A20 and C/EBPβ, indicating the potential anti-NF-κB value of endoplasmic reticulum stress in endometriosis 49.

### Oxidative stress

**Figure 3:**
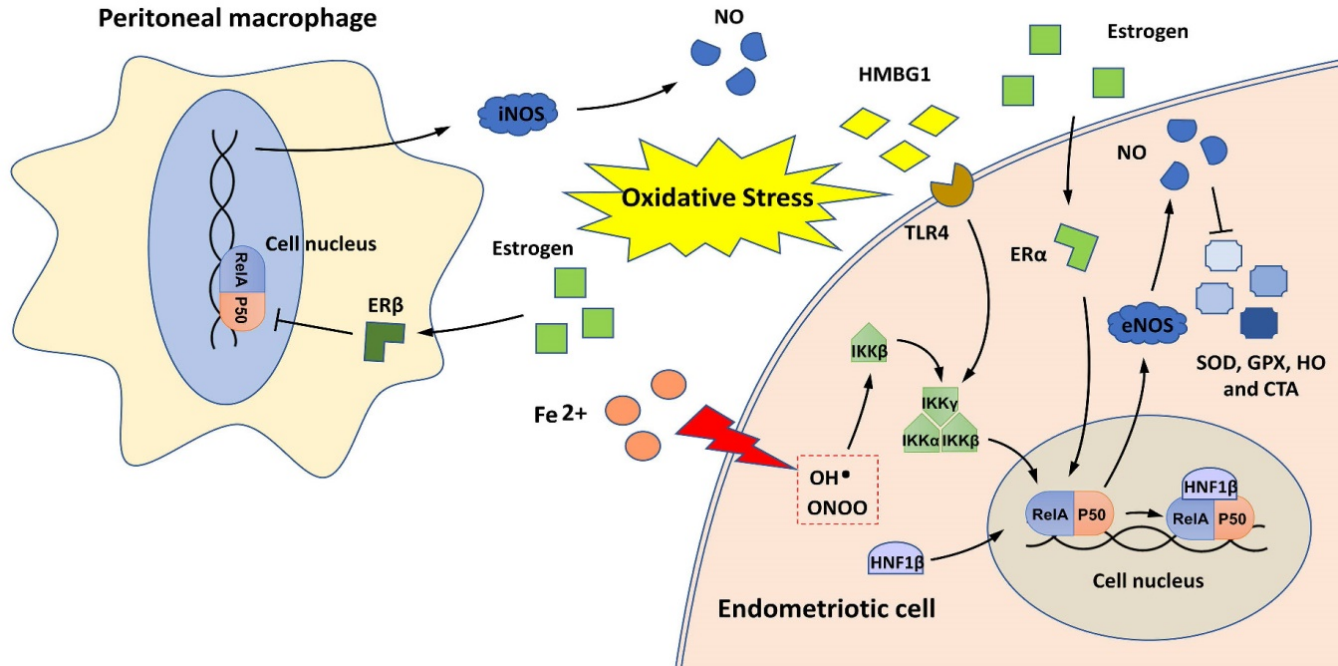
NF-κB and oxidative stress in endometriosis. Oxidative stress induces NF-κB activation in endometriotic cells through iron overload and by activating the HMGB-1/TLR4 and estrogen/ERα pathways. HNF1β, as a coactivator for NF-κB, can activate NF-κB to protect endometriotic cells against oxidative damage. NF-κB activation in endometriotic cells may also induce the production of pro-oxidants (iNOS and NO) and reduce antioxidant enzymes (SOD, GPx, HO, and CAT). In peritoneal macrophages, estrogen/ERβ signaling can inhibit the NF-κB/NOS/NO axis and may alleviate oxidative stress.

The relationship between NF-κB and oxidative stress in endometriosis is shown in **Figure 3**. The imbalance between pro-oxidants (free radical species, such as reactive oxygen species [ROS] and nitric oxide synthase [NOS] and antioxidants is implicated in the pathophysiology of endometriosis 50. The overproduction of ROS in the pelvic cavity of patients with endometriosis is an important inducer of chronic NF-κB-mediated inflammatory responses 51-53. Extracellular high mobility group box-1 (HMGB-1), a prototypical molecule of damage-associated molecular patterns, activates NF-κB in endometriotic cells by binding to its receptor, Toll-like receptor 4 (TLR4), and induces inflammatory responses in the environment of endometriosis with sustained oxidative stress 52, 53. In addition, the HMGB-1/TLR4/NF-κB axis can also induce the proliferation and invasion of endometriotic cells and contribute to endometriosis-induced pain 52-54.

During retrograde menstruation, erythrocytes are carried into the pelvic cavity of patients with endometriosis, and the lysis of erythrocytes results in iron release, with free iron serving as a source of ROS 55. Iron overload activates IKKβ and stimulates NF-κB signaling, conferring pro-endometriotic behaviors on endometrial stromal cells 27. Hepatocyte nuclear factor-1 beta (HNF1β) is a homeobox transcription factor that is overexpressed in endometriotic cells 56. It functions as a coactivator for NF-κB, and its activation can enhance the survivability of endometriotic cells in oxidative cellular environments 56.

NF-κB signaling may also contribute to oxidative stress overload in patients with endometriosis by promoting NOS production and decreasing the expression of antioxidant enzymes, such as superoxide dismutase (SOD), glutathione peroxidase (GPx), heme oxygenase (HO), and catalase (CAT) 35, 57, 58. Notably, two estrogen receptors (ERα and ERβ) are engaged in the regulation of the NF-κB/NOS/NO axis; ERα activates this axis in endometrial stromal cells, and ERβ inhibits this axis in PMs 35, 57. These findings indicate that estrogen signaling may have different effects on NF-κB-mediated oxidative stress in the ectopic endometrium and endometriotic milieu.

### Noncoding RNAs

NcRNAs are a heterogeneous class of RNAs that do not encode proteins but participate in the pathophysiological processes of endometriosis by regulating gene expression 59. MiRNAs are ncRNAs that are 19-25 nucleotides in length and negatively regulate gene expression by binding to the 3′-untranslated regions of target mRNAs 59. Four types of miRNAs, namely, miR-16, miR-138, miR-182, and miR-199a, directly target the key NF-κB signaling-related genes* IKKβ* (targeted by miR-138 and miR-182) and *p65* (targeted by miR-16 and miR-199a) to inhibit NF-κB signaling, and their expression is downregulated in ectopic endometrium 60-63. In contrast, two miRNAs, namely, miR-9 and miR-22, indirectly activate NF-κB signaling by repressing the expression of sirtuin 1 (SIRT1), an NAD (+)-dependent deacetylase, to reduce inflammatory responses in the ectopic endometrium 64, 65. The overexpression of miR-16, miR-138, miR-182, and miR-199a or the inhibition of miR-9 and miR-22 can block NF-κB signaling and further repress inflammatory responses and the survival, migration, and invasion abilities of endometriotic cells 60-65.

Long noncoding RNAs (lncRNAs) are another group of ncRNAs more than 200 nucleotides in length 59. MALAT1, a lncRNA, is overexpressed in the ectopic endometrium and enhances the proliferation and invasion of endometriotic cells by activating the NF-κB/iNOS/MMP9 axis 58. However, the mechanism by which MALAT1 activates NF-κB remains unclear.

## Role of NF-κB in endometriosis pathogenesis

### NF-κB regulates endometriotic cell behaviors

NF-κB signaling affects endometriosis progression by regulating the activities of endometriotic cells. The abnormal survivability of endometriotic cells is correlated with NF-κB-mediated activities, such as secretion of the proinflammatory cytokine IL8 and activation of the antiapoptotic molecules XIAP, Bcl-2, and Bcl-xL 18, 56, 66, 67. In a *Macaca fascicularis* model of endometriosis, *p65* knockdown by short hairpin RNA considerably reduced the expression of proliferating cell nuclear antigen (PCNA) and the microvessel density of ectopic lesions, indicating that NF-κB can be a therapeutic target for preventing the growth and angiogenesis of endometriotic lesions 68.

The aberrant adhesion of endometriotic cells is an initial step for the establishment of endometriosis 69. Decoy receptor 3 (DcR3), a pleiotropic immunomodulator, can move retrograde to the ectopic endometrium with menstrual blood and subsequently induce upregulation of adhesion molecules in an NF-κB-dependent manner 70. NF-κB signaling induces the high expression of key adhesion molecules in the ectopic endometrium, including homing cell adhesion molecule (CD44), ICAM-1, and vascular cell adhesion molecule-1 (VCAM-1). Inhibition of NF-κB can effectively reduce the adhesive ability of endometriotic cells 19, 70, 71.

The high migration and invasion ability of endometriotic cells is considered the main cause of implantation and extension of the ectopic foci 72. Studies have revealed that NF-κB signaling contributes to endometriotic cell migration and invasion through the transcriptional activation of matrix metalloproteinases (MMPs, especially MMP-2 and MMP-9), a family of zinc-dependent endopeptidases that are responsible for extracellular matrix degradation 71, 73-75. Nasiri et al. exhibited potent inhibitory effects of the anti-NF-κB drugs aloe-emodin and aspirin on the invasion of endometriotic cells from patients with stage IV endometriosis 71. In another study, *IKKβ* knockdown via short interfering RNA remarkably suppressed the migration and invasion of endometriotic cells, further indicating the critical role of NF-κB signaling in ectopic endometrial implantation 60.

### NF-κB and macrophages in the endometriotic milieu

**Figure 4:**
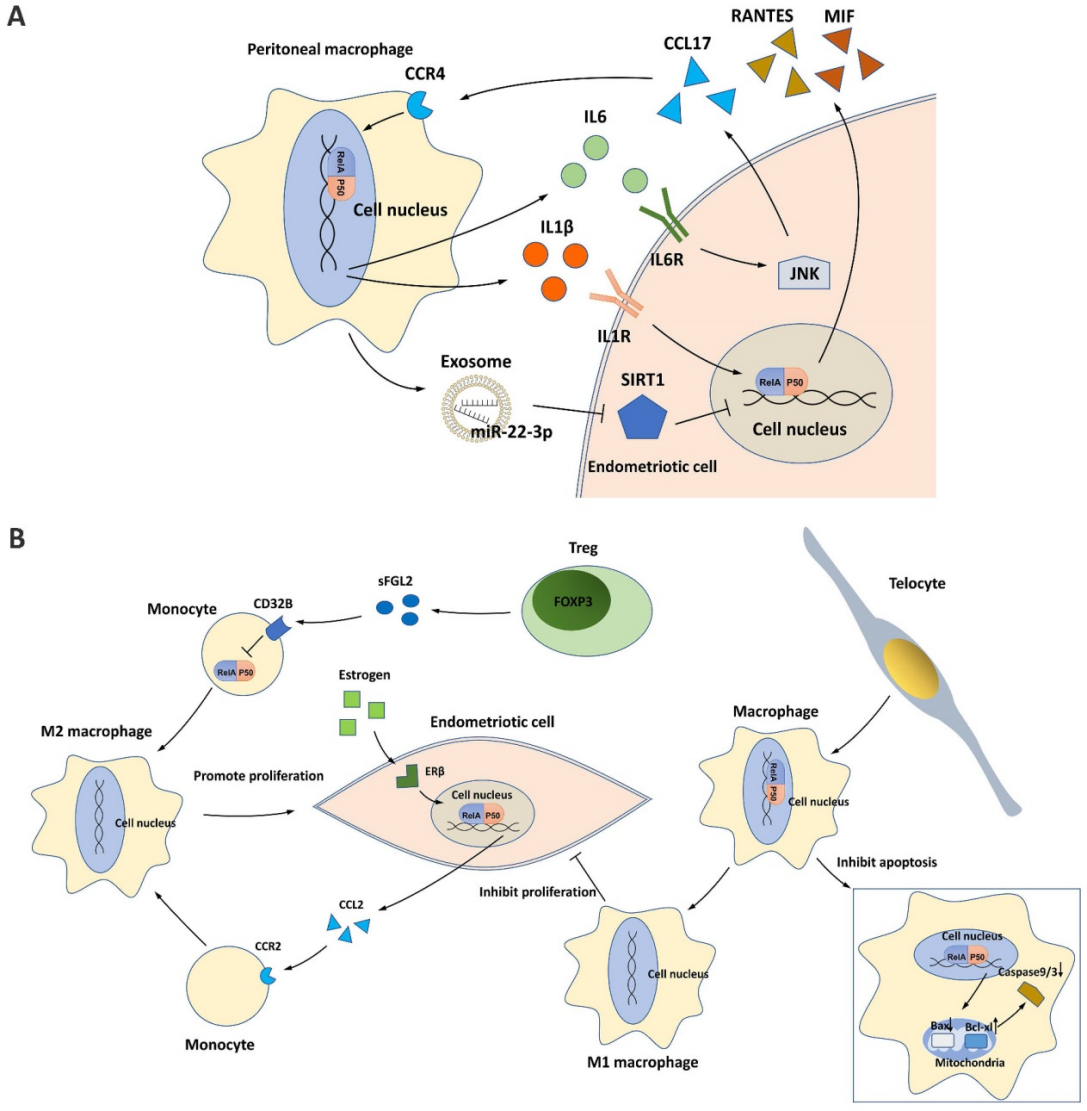
NF-κB and macrophages in endometriosis. **(A)** NF-κB signaling contributes to the crosstalk between peritoneal macrophages (PMs) and endometriotic cells. The IL6/JNK/CCL17/CCR4 axis induces NF-κB activation in PMs, which then promotes IL6 production and forms a positive loop. In addition, IL1β and exosome-derived miR-22-3p secreted by PMs induce NF-κB activation in endometriotic cells, which may promote secretion of the proinflammatory cytokines RANTES and MIF. **(B)** NF-κB signaling affects PM polarization in the endometriotic milieu. Tregs and estrogen/ERβ signaling induce M2 macrophage polarization and endometriotic cell proliferation by repressing NF-κB in monocytes and activating NF-κB in endometriotic cells. Telocytes promote M1 macrophage polarization while inhibiting endometriotic cell proliferation and mitochondria-mediated PM apoptosis by activating NF-κB in PMs.

Activated PMs are involved in the pathological process of peritoneal endometriotic lesions 76. In a study of 44 cases (22 with and 22 without endometriosis), a significantly higher proportion of NF-κB nuclear translocation was found in PMs from patients with endometriosis 77. NF-κB activation contributes to the crosstalk between PMs and endometriotic cells and affects the polarization/differentiation of PMs in the ectopic milieu **(Figure 4)**.

### Crosstalk between PMs and endometriotic cells

Endometriotic cells can activate NF-κB signaling in PMs by secreting CCL17, and this process is dependent on JNK signaling 78. NF-κB activation in PMs subsequently induces the secretion of IL6, which in turn activates the JNK/CCL17 axis in endometriotic cells, forming crosstalk between PMs and endometriotic cells 78.

PMs can also activate NF-κB signaling in endometriotic cells through different mechanisms. IL1β, a potent macrophage cytokine produced from activated PMs in the ectopic milieu, activates NF-κB in endometriotic cells, resulting in the production of proinflammatory cytokines/chemokines, such as RANTES and MIF 13, 14, 21. In addition, PMs can release exosomes that deliver miR-22-3p, a miRNA that activates NF-κB signaling in endometriotic cells by suppressing SIRT1 expression 65.

### Macrophage polarization

Traditionally, macrophages differentiate into classical proinflammatory M1 macrophages or alternative anti-inflammatory M2 macrophages in response to different environmental stimuli. In the endometriotic milieu, M1 macrophages secrete multiple cytokines/chemokines for inflammatory responses, inhibiting endometriotic cell proliferation and promoting tissue damage, whereas M2 macrophages possess an immunosuppressive ability that supports the survival and invasiveness of endometriotic cells, stimulates the growth and vascularization of ectopic endometrial lesions, and induces pain generation 78-82. During the progression of endometriosis, NF-κB suppression in monocytes/macrophages enhances M2 macrophage polarization and inhibits M1 macrophage polarization, developing a pro-repair environment for neovascularization in ectopic lesions 80, 81. One mechanism is the binding of soluble fibrinogen-like protein 2 secreted by the increased regulatory T cells (Tregs) in the endometriotic milieu to its receptor CD32B expressed on PMs 81. Tregs activated by PMs further suppress NF-κB signaling and induce an immune tolerance environment for endometriosis progression 81. Telocytes, a type of mesenchymal/stromal cell, were recently identified to enhance M1 macrophage polarization in the endometriotic milieu by activating NF-κB signaling, which helps suppress the onset of endometriosis 80. In addition, NF-κB activated by telocytes can also promote macrophage proliferation by inhibiting mitochondrion-dependent apoptosis 80.

In endometriotic cells, NF-κB signaling is activated by estrogen/ERβ signaling and can enhance M2 macrophage polarization 32. Mechanistically, estrogen-stimulated NF-κB signaling promotes CCL2 production, which recruits PMs and induces macrophage M2 polarization, thus promoting the pathogenesis of endometriosis 32.

### NF-κB contributes to endometriosis-associated pain and infertility

#### NF-κB and pain

The presence of TRPA1/TRPV1-expressing nerve fibers in ectopic endometrium is one of the key factors for pain generation 83. In a C57BL/6 mouse model of endometriosis, Fattori et al. observed that endometriosis-induced NF-κB activation contributes to increased calcium influx in TRPA1/TRPV1-expressing dorsal root ganglion (DRG) neurons 84. This pattern of neuronal activation coincides with peripheral sensitization detected in the activation of NF-κB 85, 86.

In an SD rat model of endometriosis, NF-κB overexpression was found in the DRG and spinal dorsal horn (SDH), which was induced by the HMGB-1/TLR4/MyD88 pathway and contributed to mechanical hyperalgesia at the graft site of ectopic endometrium 54. Inhibiting the expression of TLR4 or MyD88 could decrease NF-κB p65 phosphorylation in the DRG, alleviating chronic endometriosis-induced pain 54.

Nobiletin and andrographolide, which are plant-derived anti-NF-κB drugs, can remarkably improve response latency in animal models of endometriosis, confirming the potential of NF-κB as a target for reducing endometriosis-induced pain 87, 88.

#### NF-κB and infertility

In a study of 35 cases (15 infertile patients with endometriosis, 10 infertile patients with nonendometriotic ovarian cysts, and 10 healthy fertile women), the mean expression of NF-κB1 was remarkably higher in the ectopic endometrium of infertile patients with endometriosis than in the endometria of patients with nonendometriotic cysts and fertile patients 89. After the surgical removal of endometrioma, the expression of NF-κB1 markedly decreased in endometriosis patients, suggesting that the overexpression of NF-κB in eutopic endometrium may contribute to endometriosis-associated infertility 89. According to the results of another genetic association study of 438 cases (172 infertile patients with endometriosis, 77 cases of idiopathic infertility, and 189 healthy women), the 94 insertion/deletion ATTG polymorphism in the *NFKB1* gene was positively correlated with endometriosis and idiopathic infertility 90. More investigations should be performed to explore the relationship between NF-κB activation and the high infertility risk of patients with endometriosis.

## Preclinical drugs for blocking NF-κB signaling in endometriosis

Preclinical *in vivo* and/or *in vitro* experiments have discovered a large number of drugs that inhibit NF-κB signaling and thus alleviate the development of endometriosis **(Table 1)** 13, 14, 16, 18, 19, 36, 46, 67, 75, 87, 88, 91-119. In this section, we discuss the effects of some of these drugs on endometriotic cells/endometriosis-like lesions and the relevant mechanisms. We also predicted potential drugs that may affect NF-κB signaling in endometriosis through the Drug-Gene Interaction Database (DGIdb) (http://www.dgidb.org/) 120 to provide more potential drug treatment options for researchers.

### Plant-derived medicines

Numerous studies have shown that plant extracts are a source of novel therapeutic methods for endometriosis 121, and NF-κB signaling was identified as the target of some of these plant-derived medicines 13, 14, 16, 19, 67, 87, 88, 91-100, 117, 118. Curcumin derived from the rhizomes of *Curcuma* plants can repress TNFα/IL1β-induced NF-κB activation in human endometriotic cells, resulting in the reduced secretion of proinflammatory cytokines, such as IL6, IL8, and MIF, and the reduced expression of chemokines, such as MCP-1, and cell adhesion molecules, such as ICAM-1 and VCAM-1 13, 14, 19. In addition, *in vivo* experiments revealed that curcumin can also inhibit MMP3-dependent FasL-induced local immune cell death in the endometriotic milieu partly by suppressing NF-κB activation, which prevents the formation of the immune-tolerant environment in initial endometriotic development 99.

Andrographolide, an active ingredient extracted from *Andrographis paniculate*, is a potent NF-κB inhibitor in endometriosis. Mechanistically, andrographolide attenuates the DNA-binding activity of NF-κB and the expression of its downstream genes COX-2, TF, and NGF, suppressing the proliferation of endometriotic cells and reducing the size of ectopic lesions 88. A recent study also found that the perioperative use of β-blockers and/or andrographolide can effectively inhibit the growth of residual lesions in a BALB/c mouse model of endometriosis, indicating the potential value of andrographolide in reducing the recurrence risk of endometriosis 100.

In addition to curcumin and andrographolide, other plant-derived medicines, such as ginsenoside Rg3, baicalein, costunolide, imperatorin, 6-shogaol, octyl gallate, parthenolide, HEABG, nobiletin, Artemisia princeps extract, glycyrrhizin, and Cypri rhizoma extract, also inhibit inflammation and endometriotic cell proliferation by suppressing NF-κB signaling 16, 67, 87, 91-97, 117, 118. However, the exact mechanisms need further exploration.

### NF-κB inhibitors

BAY 11-7085 is a synthetic compound that suppresses IκBα phosphorylation and prevents the release and nuclear translocation of NF-κB 122. BAY 11-7085 treatment can remarkably inhibit DNA synthesis and the proliferation of endometriotic cells and induce cell apoptosis by activating caspase-mediated apoptosis 101. SN50, a cell-permeable NF-κB inhibitory peptide, consists of a membrane-translocating motif and a nuclear localization sequence derived from the NF-κB p50 subunit, which specifically inhibits the nuclear translocation of NF-κB 123. NF-κB inhibition through BAY 11-7085 or SN50 treatment reduced endometriotic lesions and diminished the initial development of endometriosis in a nude mouse model of endometriosis 102.

Pyrrolidine dithiocarbamate (PDTC), a diethyl derivative of dithiocarbamates, is another potent NF-κB inhibitor. It inhibits NF-κB signaling by repressing IκBα phosphorylation, nuclear p65 protein expression, and the DNA-binding activity of NF-κB subunits in endometriotic cells 105. The expression of NF-κB target genes/molecules in endometriotic cells is also inhibited by PDTC treatment, which may suppress the proliferation (PCNA, CD31, CD34, Ki67, and survivin), angiogenesis (VEGF), adhesion (CD44), and migration/invasion (MMP2, MMP9) of endometriotic cells and reduce inflammatory responses (COX-2, PGE2) 75, 103-105.

### Other known drugs

Pioglitazone is a peroxisome proliferator-activated receptor γ ligand that can inhibit TNFα-induced IL-8 expression and endometriotic cell proliferation by suppressing NF-κB signaling 18. Notably, activation of the TNFα/NF-κB/IL8 pathway in endometriotic cells can also be inhibited by hormone or thalidomide treatment, providing other choices for blocking NF-κB signaling in endometriosis 46, 108, 109. BV6 is a small-molecule antagonist of inhibitors of apoptosis proteins (IAPs) that are activated by NF-κB signaling in endometriosis. BV6 causes the proteasomal degradation of IAPs and suppresses their expression; thus, it inhibits endometriotic cell proliferation *in vitro* and the growth and inflammation of murine endometriosis-like lesions *in vivo* 110, 111.

Niclosamide is an antihelminthic drug used to treat parasitic infections. Recent studies have demonstrated that niclosamide can suppress macrophage-dependent endometriotic cell viability and cytokine/chemokine secretion through STAT3 and NF-κB signaling, but its therapeutic effect needs to be verified in animal models of endometriosis 114, 115.

### Potential drugs

**Figure 5:**
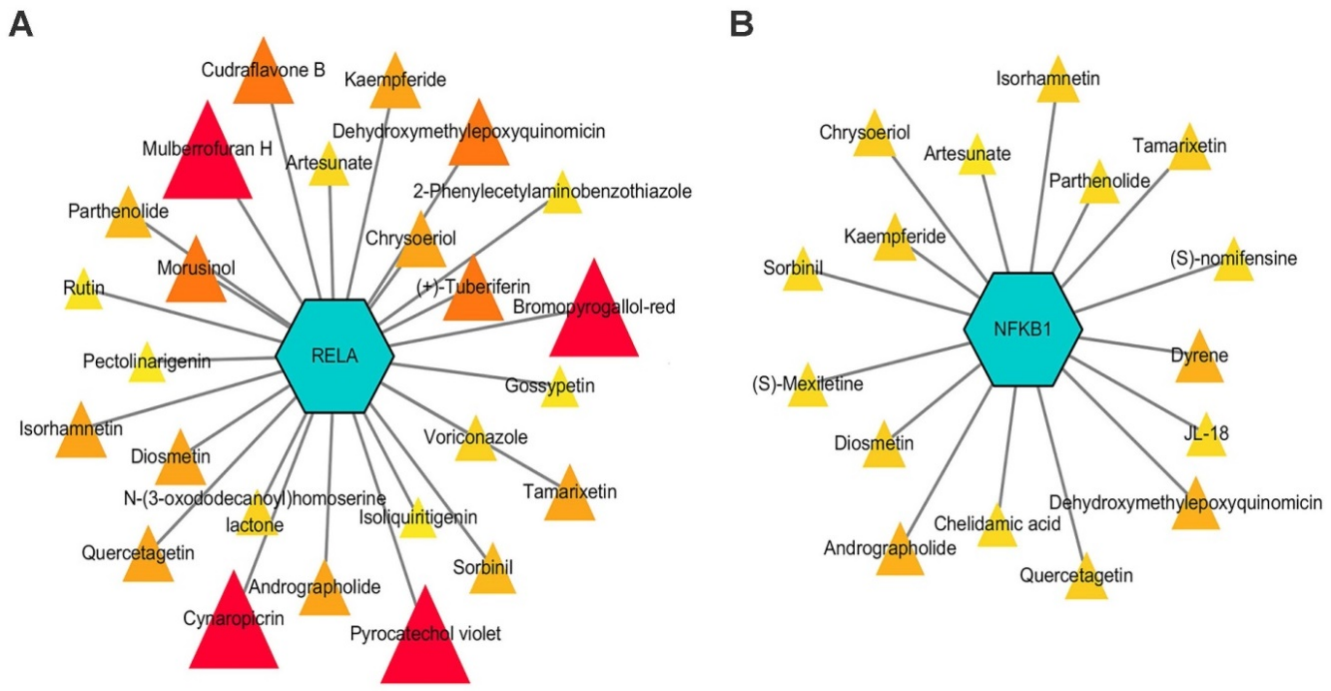
Potential interactions between drugs and key NF-κB signal-related genes, *RELA* and *NFKB1*. Drugs with an interaction score ≥ 0.2 were screened out. Triangles with sizes from small to large and colors from light to dark represent interaction scores from low to high.

The druggability of NF-κB signaling-related genes was described using DGIdb, and potential drug-gene interactions were visualized using Cytoscape 124. The results showed that *RELA* and *NFKB1*, two key NF-κB signaling-related genes, were theoretically regulated by 25 and 16 drugs, respectively **(Figure 5)**. Among these drugs, four NF-κB inhibitors, namely, isoliquiritigenin, isorhamnetin, parthenolide, and rutin, prevented inflammatory responses and inhibited the development of endometriosis in preclinical studies 97, 125-127. Other drugs, including dehydroxymethylepoxyquinomicin, edasalonexent, acacetin, artesunate, chrysoeriol, cudraflavone B, cynaropicrin, N-(3-oxododecanoyl) homoserine lactone, quercetagetin, sorbinil, tamarixetin, diosmetin, laquinimod, and triptolide, also have inhibitory effects on the NF-κB signaling-induced inflammatory response; thus, their effects on endometriosis need further exploration 128-136.

## Discussion and Conclusion

Stimulated by proinflammatory factors, such as IL1β and TNFα, NF-κB signaling is overactive in ectopic endometrial tissues and enhances the proliferation, adhesion, migration, and invasion abilities of endometriotic cells. In addition, NF-κB signaling is involved in the crosstalk between endometriotic cells and PMs, as well as the regulation of PM polarization to affect endometriosis progression.

Estrogen plays a key role in regulating NF-κB signaling in endometriosis and has promoting and inhibitory effects. Progesterone has a clear inhibitory effect on NF-κB activity, and hormone therapies, such as GnRHa and progesterone treatment, can remarkably inhibit NF-κB-related inflammatory responses in endometriotic cells.

Oxidative stress is a key inducer of NF-κB signaling in endometriotic cells by activating the HMGB-1/TLR4/NF-κB axis and inducing iron overload. NF-κB signaling can in turn contribute to oxidative stress by activating NOS/NO signaling and decreasing the expression of antioxidant enzymes. NcRNAs, such as miRNAs and lncRNAs, are abundantly found in the human endometrium and were recently identified to regulate the expression of NF-κB-related genes in endometriotic cells.

In patients with endometriosis, the activation of NF-κB signaling is associated with endometriosis-related pain and infertility. In preclinical studies of endometriosis, plant-derived drugs, NF-κB inhibitors, and other known drugs have been widely evaluated and have shown potent anti-NF-κB effects that alleviate disease progression. Other drugs that have the potential to target NF-κB-related molecules are predicted in this review using DGIdb.

Notably, most NF-κB-related endometriosis studies chose normal/ectopic endometrial stromal cells for subsequent experiments. However, several studies also revealed that the role of NF-κB in endometrial epithelial cells shares several similarities with its role in endometrial stromal cells, such as the regulation by TNFα, estrogen signaling, and ncRNAs 24, 31, 61; the promotion of cell proliferation, migration, invasion, and adhesion 56, 73, 74; and the contribution to the resistance of ROS stress-induced cell apoptosis 56. In addition, several drugs exert anti-endometriotic effects by downregulating NF-κB expression in endometrial epithelial cells 67, 93, 117-119.

In conclusion, aberrant NF-κB signaling is involved in several aspects of endometriosis pathogenesis. Future studies should discover and develop more drugs that inhibit NF-κB signaling and further test their efficacy in clinical trials.

## Figures and Tables

**Table 1:** Known drugs that target NF-κB signaling in endometriosis.

Drug	Function	Experimental model/condition	Reference
Plant/herbal extract			
Baicalein	Inhibits endometriotic cell viability via suppressing NF-κB signaling.	hESCs isolated from patients with endometriosis	92
Imperatorin	Inhibits the growth and the histopathological features of endometriosis-like lesions via suppressing PI3K/Akt/NF-κB pathway.	SD rat model	94
6-Shogaol	Inhibits inflammation of endometriosis-like lesions via suppressing NF-κB signaling.	SD rat model	95
Octyl Gallate	Inhibits inflammation of endometriosis-like lesions via suppressing NF-κB signaling.	Wistar rat model	96
HEABG; Ginsenoside Rg3	Inhibits endometriotic cell proliferation via suppressing TNFα/NF-κB pathway*.*	hESCs isolated from patients with endometriosis	16, 91
Parthenolide	Reduces lesion size and growth of endometriosis-like lesions via suppressing TNFα/NF-κB pathway.	BALB/c mouse model	97
Curcumin	Inhibits inflammation of endometriosis-like lesions partly via suppressing NF-κB signaling.	hESCs isolated from patients with endometriosis	98
	Inhibits the secretion of MIF in hESCs via suppressing IL-1β/NF-κB pathway.	hESCs isolated from patients with endometriosis	13, 14
	Inhibits the expression of IL-6, IL-8, MCP-1, ICAM-1 and VCAM-1 in hESCs via suppressing TNFα/NF-κB pathway.	hESCs isolated from patients with endometriosis	19
	Ameliorates decreased apoptotic responses during early endometriosis caused partly via suppressing NF-κB/MMP-3/FasL pathway.	BALB/c mouse model	99
Nobiletin	Reduces lesion sizes, pain and inflammation of endometriosis-like lesions via suppressing NF-κB signaling.	hESCs isolated from patients with endometriosis; C57BL/6 mouse model	87
Andrographolide	Reduces lesion size and growth of endometriosis-like lesions and improves hyperalgesia via suppressing NF-κB signaling.	hESCs isolated from patients with endometriosis; SD rat model	88
	Reduces the recurrence of endometriosis partly via suppressing NF-κB signaling.	BALB/c mouse model	100
Glycyrrhizin	Inhibits the production of LPS-induced inflammatory mediators via suppressing NF-κB signaling.	Primary mouse endometrial epithelial cells	117
Costunolide	Promotes endometriotic cell apoptosis via suppressing NF-κB signaling.	Human endometriotic epithelial cell line 11Z	93
Artemisia princeps extract	Promotes endometriotic cell apoptosis via suppressing NF-κB signaling.	Human endometriotic epithelial cell line 11Z and 12Z	67
Cyperi rhizoma extract	Inhibits endometriotic cell adhesion and neurotrophin expression via suppressing Akt/NF-kB pathway.	Human endometriotic epithelial cell line 11Z and 12Z	118
NF-κB inhibitor			
BAY 11-7085	Inhibits endometriotic cell proliferation via suppressing NF-κB signaling.	hESCs isolated from patients with endometriosis	101
BAY 11-7085 and SN-50	Promotes the apoptosis of endometriosis-like lesions via suppressing NF-κB signaling.	Nude mouse model of endometriosis	102
PDTC	Inhibits proliferation, angiogenesis, adhesion, migration and invasion of endometriotic cells via suppressing NF-κB signaling.	hESCs isolated from patients with endometriosis	75, 103-105
Other known drugs			
hCG; Daidzein-rich isoflavone aglycones	Suppresses TNFα/NF-κB pathway in endometriotic cells.	hESCs isolated from patients with endometriosis	106, 107
Progesterone, dienogest, or danazol; GnRHa; Thalidomide	Suppresses TNFα/NF-κB/IL8 pathway in endometriotic cells.	hESCs isolated from patients with endometriosis	46, 108, 109
Pioglitazone	Inhibits endometriotic cell proliferation via suppressing TNFα/NF-κB/IL-8 pathway.	hESCs isolated from patients with endometriosis	18
BV6	Inhibits endometriotic cell viability via suppressing TNFα/NF-κB/cIAPs pathway.	hESCs isolated from patients with endometriosis	110
	Reduces endometriotic lesion size and represses the inflammatory and angiogenic activity of the endometriosis-like lesions via suppressing NF-κB/cIAPs pathway.	BALB/c mouse model	111
CDDO-Me	Inhibits inflammation in endometriosis-like lesions via suppressing NF-κB signaling.	SD rat model	112
Disulfiram	Prevents endometriotic implant growing via suppressing NF-κB signaling.	Wistar rat model	113
SR-16234	Inhibits the growth of endometriosis-like lesions partly via suppressing NF-κB signaling.	BALB/c mouse model	36
INT-777	Suppresses TGR5/TNFα/NF-κB pathway in endometriotic cells.	hESCs isolated from patients with endometriosis	116
Niclosamide	Inhibits macrophage-induced inflammation and endometriotic cell viability partly via suppressing NF-κB signaling.	Human endometriotic epithelial cell line 12Z	114
		hESCs isolated from patients with endometriosis	115
Trichostatin A	Suppresses TNFα/NF-κB pathway in endometriotic cells.	Human endometriotic stromal cell line YHES, and 22B; Human endometriotic epithelial cell line 11Z	119

hESC: human endometrial stromal cell; PI3K: phosphatidylinositol 3 kinase; Akt: protein kinase B; SD: Sprague Dawley; HEABG: hexane extract of aged black garlic; TNFα: tumor necrosis factor alpha; IL: interleukin; MCP-1: monocyte chemoattractant protein 1; ICAM-1: intercellular adhesion molecule 1; VCAM-1: vascular cell adhesion molecule 1; MMP: matrix metalloproteinase; PDAC: pyrrolidine dithiocarbamate; LPS: lipopolysaccharide; TGR5: takeda-G-protein-receptor-5

## Abbreviations

NF-κB: nuclear factor-kappa B
IL1: interleukin1
IL6: interleukin 6
IL8: interleukin 8
RANTES: regulated on activation normal T cell expressed and secreted
MIF: macrophage migration inhibitory factor
ER: estrogen receptor
CXCL12: C-X-C motif chemokine 12
CXCR4: C-X-C chemokine receptor type 4
PI3K/Akt: phosphoinositide 3-kinase/ protein kinase B
PTEN: phosphatase and tensin homolog
MAPK/ERK: mitogen-activated protein kinases/extracellular signal-regulated kinase
GnRHa: gonadotrophin-releasing hormone analogues
NAD: nicotinamide adenine dinucleotide
XIAP: X-linked inhibitor of apoptosis
Bcl-2: B-cell lymphoma 2
Bcl-xL: B-cell lymphoma-extra-large
JNK: jun N-terminal kinase
CCL17: C-C motif chemokine 17
CCL 2: C-C motif chemokine 2
TRPA1: transient receptor potential ankyrin 1
TRPV1: transient receptor potential vanilloid-1
MYD88: myeloid differentiation factor-88 adaptor protein
MCP1: monocyte chemoattractant protein 1
TF: tissue factor
NGF: nerve growth factor
COX2: cyclooxygenase-2
